# MicroRNA‐based risk scoring system to identify early‐stage oral squamous cell carcinoma patients at high‐risk for cancer‐specific mortality

**DOI:** 10.1002/hed.26089

**Published:** 2020-01-25

**Authors:** Angela J. Yoon, Shuang Wang, David I. Kutler, Richard D. Carvajal, Elizabeth Philipone, Tian Wang, Scott M. Peters, Dominic LaRoche, Brenda Y. Hernandez, Bradley D. McDowell, Claire R. Stewart, Fatemeh Momen‐Heravi, Regina M. Santella

**Affiliations:** ^1^ Division of Oral and Maxillofacial Pathology, Department of Pathology & Cell Biology Columbia University College of Dental Medicine, Columbia University Irving Medical Center New York New York; ^2^ Department of Biostatistics Columbia University Mailman School of Public Health New York New York; ^3^ Department of Otolaryngology‐Head and Neck Surgery Weill Cornell Medical College New York New York; ^4^ Department of Medical Hematology and Oncology Columbia University Irving Medical Center New York New York; ^5^ HTG Molecular Diagnostic, Inc. Tucson Arizona; ^6^ Hawaii Tumor Registry, University of Hawaii Cancer Center Honolulu Hawaii; ^7^ Holden Comprehensive Cancer Center, University of Iowa Iowa City Iowa; ^8^ Division of Periodontics Columbia University College of Dental Medicine New York New York; ^9^ Department of Environmental Health Sciences Columbia University Mailman School of Public Health New York New York

**Keywords:** deep sequencing, microRNA, oral squamous cell carcinoma, prognostic marker, risk score

## Abstract

**Background:**

For early‐stage oral squamous cell carcinoma (OSCC), there is no existing risk‐stratification modality beyond conventional TNM staging system to identify patients at high risk for cancer‐specific mortality.

**Methods:**

A total of 568 early‐stage OSCC patients who had surgery only and also with available 5‐year clinical outcomes data were identified. Signature microRNAs (miRNAs) were discovered using deep sequencing analysis and validated by qRT‐PCR. The final 5‐plex prognostic marker panel was utilized to generate a cancer‐specific mortality risk score using the multivariate Cox regression analyses. The prognostic markers were validated in the internal and external validation cohorts.

**Results:**

The risk score from the 5‐plex marker panel consisting of miRNAs‐127‐3p, 4736, 655‐3p, TNM stage and histologic grading stratified patients into four risk categories. Compared to the low‐risk group, the high‐risk group had 23‐fold increased mortality risk (hazard ratio 23, 95% confidence interval 13‐42), with a median time‐to‐recurrence of 6 months and time‐to‐death of 11 months (vs >60 months for each among low‐risk patient; *p* < .001).

**Conclusion:**

The miRNA‐based 5‐plex marker panel driven mortality risk score formula provides clinically practical and reliable measures to assess the prognosis of patients assigned to an early‐stage OSCC.

## 1 INTRODUCTION

An estimated 30 000 people in the US are diagnosed with oral squamous cell carcinoma (OSCC) each year.^1^, ^2^, ^3^, ^4^ OSCC is a deadly disease, accounting for 7400 deaths each year in the US.^4^, ^5^ Of the newly diagnosed oral cancer cases, 50% are in tumor‐node‐metastasis (TNM) stage I/II without regional lymph node involvement or distant metastasis.^2^

While the TNM stage is considered to be the key prognostic determinant in oral cancer,^6^, ^7^, ^8^, ^9^, ^10^, ^11^, ^12^, ^13^, ^14^ it is incapable of delineating individual risk for patients within the same TNM stage strata. For example, if 10 patients with similar demographics present with ~3 cm OSCC of the tongue with the depth of invasion of 4 mm, without clinical or radiographic evidence of positive cervical lymph node or distant metastasis, all 10 patients will be assigned to TNM stage II. Out of these 10 patients, four will die of cancer (28%‐42% 5‐year mortality rate for early‐stage oral cancer patients).^7^ Currently, there is no existing risk‐stratification modality beyond the conventional TNM staging system to identify those four patients at high‐risk for cancer‐specific death.

There is a critical need to stratify traditional tumor classes into subsets that behave differently from each other to refine and improve prognostication and treatment selection.^15^ The TNM staging system is based on anatomic extent of disease, and is determined by tumor size (T), affected regional lymph nodes (N), and distant metastases (M), which are the TNM variables.^6^, ^7^, ^8^, ^9^, ^10^, ^11^, ^12^, ^13^, ^14^ The American Joint Committee on Cancer (AJCC) revised the staging system (eighth edition, 2017), in which the depth of invasion (DOI) was added as a modifier for the T category (T1 = size ≤2 cm and DOI ≤ 5 mm; T2 = size ≤ 2 cm and DOI > 0.5 but ≤ 1.0 cm, or size 2‐4 cm and DOI ≤ 1.0 cm; T3 = size > 4 cm or DOI > 1.0 cm).^7^, ^8^, ^9^ The AJCC 8 staging system performs better in stratifying survival of OSCC patients by stage.^10^, ^11^ For early stage OSCC with clinically negative lymph nodes, greater DOI is associated with increased risk of occult lymph node metastasis.^12^, ^13^ Such finding may be due to increased chance of perineural invasion and close/positive deep margins in tumors with greater DOI, resulting in regional metastasis.^12^, ^13^ With restaging, the survival concordance index improved from 0.699 to 0.704 (from seventh to eighth edition, respectively).^7^

However, carcinogenesis is not solely defined by the TNM stage the patient is in at the time of diagnosis but also heavily influenced by the molecular (genomic and proteomic) characteristics of the tumor.^16^, ^18^ For this reason, consideration of biological determinism reflected in altered expression of biomarkers, in addition to anatomic extent of disease is becoming increasingly important in personalized medicine.^17^ Since biomarkers are interacting, interdependent, multipurpose parts of the biological system, the prognostic model consisting of multiple markers can increase the predictive power and capture the clinical behavior of cancer better than a single marker.^18^ In concordance with this concept, we developed a novel microRNA‐based prognostic model to predict the survival outcome of patients who were already categorized into “early‐stage” by the TNM system. This five‐plex marker panel considers both the anatomic extent of disease, as well as the biology of the disease itself. Subsequently, the miRNA‐based marker is well suited to sub‐risk stratify early TNM stage patients and identify the 35% of high‐risk patients (the 5‐year survival rate for early‐stage oral cancer is ~65%) who will benefit from additional treatments.

MicroRNAs (miRNAs) are small, 18‐24 nucleotide long, noncoding RNA molecules that regulate the expression of targeted genes either by facilitating mRNA degradation or by repressing translation.^19^, ^20^ One miRNA is capable of binding over 100 different mRNAs with different binding efficiencies and plays a crucial role in their posttranscriptional regulation.^19^, ^20^, ^21^, ^22^, ^23^ MicroRNAs control cell growth, apoptosis and differentiation, and various types of cancer have demonstrated distinct miRNA expression profiles.^19^, ^20^, ^21^, ^22^, ^23^ So far, a number of miRNAs associated with clinical outcomes have been reported for lung, breast, gastric and pancreatic cancers, as well as OSCC/head and neck carcinomas.^19^, ^20^, ^21^, ^22^, ^23^, ^24^, ^25^, ^26^, ^27^, ^28^, ^29^

In this study, we assessed differential expression of miRNAs genome‐wide via deep sequencing in tumor tissue samples and identified signature miRNAs that can serve as a prognostic marker of cancer survival. The prognostic model consisting of promising miRNAs and pertinent clinicodemographic covariates was subjected to stringent internal and external validation for accuracy and reliability. The mortality risk score formula was generated with the selected prognostic signatures, which can be clinically utilized to assign individual cancer‐specific mortality risk status.

## 2 MATERIALS AND METHODS

### 2.1 Subjects and study design

Following approval from the Institutional Review Board (IRB), the internal subjects from Columbia University Irving Medical Center (CUIMC), and Weill Cornell Medical Center (WCMC) within New York‐Presbyterian Hospital (NYPH) were identified, consisting of 306 early stage OSCC patients, ≥18 years old, newly diagnosed with primary OSCC and with a minimum of 5‐year clinical outcomes information. Based on the medical record, only those who underwent surgical treatment with curative intent without elective neck dissection and/or adjuvant chemo/radiotherapy between 1995 and 2012 were selected. Subjects who were found to have occult lymph node metastasis following initial surgery were excluded. Subjects with the Eastern Cooperative Oncology Group (ECOG) performance‐status score of 0 (no symptoms) or 1 (mild symptoms) were included. One hundred (100) internal subjects were randomly selected for the deep sequencing analysis. The deep sequencing analysis subjects and an additional 100 subjects randomly selected from the internal subject pool were assigned to the internal test cohort (n = 200), and the remaining to the internal validation cohort (n = 106). The external subjects were from three cancer registries (n = 262), including the National Cancer Institute (NCI)‐sponsored Residual Tissue Repository (RTR) Program at the University of Hawaii Cancer Research Center, the Iowa Cancer Registry at the University of Iowa, and the Eastern Division of Cooperative Human Tissue Network (CHTN). The cancer registry programs do not collect time‐to‐cancer recurrence information. Therefore, only overall survival (time‐to‐death) information was available as an outcome in most external cases.

The following clinicodemographic information was obtained from the electronic clinical record; age at diagnosis, gender, race/ethnicity (white non‐Hispanic, white Hispanic, black non‐Hispanic, black Hispanic, Asian), TNM stage (I vs II), histologic tumor grade (well vs moderate‐to‐poorly differentiated), close (<5 mm) or positive surgical margins, evidence of perineural invasion, depth of invasion, tobacco use (never/former vs current), and alcohol abuse (4 or more drinks on any day or 8 or more drinks per week; never/former vs current). Time of initial surgical treatment until cancer recurrence and cancer‐specific death were also noted.

For each subject, archived formalin‐fixed paraffin‐embedded (FFPE) tissue blocks were retrieved. In case the subject had recurrent and/or second primary OSCC, the initial OSCC surgical tissue sample was utilized for the analysis. Ten 10‐μm sections were obtained from archived FFPE tumor tissue samples for all subjects. For each sample, a representative section was stained with H&E and reviewed by a pathologist to identify regions containing >90% malignant epithelial cells for macrodissection. Total RNA was isolated from tissues using RNeasy FFPE kits (Qiagen Inc., Valencia, CA) following the manufacturer's protocol, yield was quantitated by Nanodrop, and samples were stored at −80°C.

### 2.2 Deep sequencing analysis

Targeted miRNA sequencing of the 100 OSCC internal subjects was performed with the HTG EdgeSeq system using the miRNA Whole Transcriptome Assay (HTG Molecular Diagnostics, Inc., Tucson, AZ).^30^ The Study Tracker randomized function was used to randomize the placement of samples and technical replicates to reduce potential intraplating biases. Tumor samples were processed as singletons and Human Brain RNA controls in triplicate. Briefly, target capture was performed by adding Nuclease Protection Probes to each sample to hybridize to the target mRNA. The library was prepared using HTG EdgeSeq PCR processing and quantified using HTG EdgeSeq KAPA Library Quantification for Illumina Sequencing. The automated HTG Library Calculator was used to ensure sufficient concentration of samples for library pooling and also to determine the appropriate dilution for the library pool. The PhiX control adaptor‐ligated library was spiked in the pooled library, which was heat denatured and loaded into the well of the NextSeq sequencing cartridge. Sequencing was performed on the Illumina NextSeq platform. The sequencing data were imported into HTG EdgeSeq Parser software to align the FASTQ files obtained from the sequencer to miRBase v.21 sequences. Post‐sequencing quality control (QC) was performed. Ninety‐one of 100 samples passed HTG post‐sequencing QC metrics. The data were deposited in the publicly available Gene Expression Omnibus (GEO) database (GSE107830).

### 2.3 MicroRNA target prediction and pathway analysis

Network visualization and functional analysis was performed using Cytoscape v3.2.0. Potential gene targets of the selected miRNAs were identified and the relative strength of the functional association between the miRNAs and the molecular pathways of those gene targets were evaluated. The pathways with log_2_ (*p*‐value) < 4.5 were selected.

### 2.4 MicroRNA expression assessment by quantitative real‐time PCR

From the deep sequencing analysis, promising prognostic miRNAs were selected and their expression levels were verified using the miScript II Rt kit (Qiagen). Briefly, 9 μL of isolated RNA was added to the cDNA master mix, composed of 5× miScript HiSpec Buffer, 10× miScript Nucleics Mix, miScript Reverse Transcriptase Mix, and water, to a total volume of 20 μL. The cDNA was incubated at 37°C for 60 minutes, followed by 5 minutes incubation at 95°C and then diluted 11 times. For amplification reactions, the miScript miRNA PCR Custom Array with a miScript SYBR Green PCR kit (Qiagen) was used in a 7300 qPCR system (Applied Biosystems, Foster City, CA), following the cycling conditions recommended by the supplier (15 minutes at 95°C, followed by 40 cycles of 15 seconds at 94°C, 30 seconds at 55°C, and 30 seconds at 70°C). The coefficient of variation was calculated and values <5% were considered acceptable. Test samples were assayed in duplicate with the laboratory blinded to survival status and with 5% duplication after relabeling. Data was analyzed to determine the threshold cycle (Ct). The endogenous control RNU6‐6p was used to normalize the relative expression of target miRNAs (ΔCt). The samples with undetermined Ct value for the control (RNU6‐6p) were excluded from analysis (nine samples from internal test cohort, four from internal validation cohort, and three from external validation cohort). Those with an undetermined Ct for specific miRNAs were assigned a value of 39.99.

### 2.5 Statistical analysis

Association between categorical patient characteristics and binary patient survival outcomes [poor outcome (cancer‐specific death within 5‐years) vs favorable outcome (5‐year survival)] were analyzed using Fisher's exact test, Pearson's chi‐squared test and the Cohchran‐Armtage trend test. From deep sequencing analysis, the differential expression of miRNAs was assessed using the edgeR analysis pipeline, which assumes a negative binomial distribution of the read counts.^31^, ^32^ We calculated the log2‐fold change of normalized expression levels of miRNAs in patients with poor prognosis vs those with favorable prognosis. The *p*‐value testing the differential expression levels of miRNAs between two prognostic groups was adjusted into a false discovery rate (FDR) following Benjamin and Hochberg method.^33^ All miRNAs with an absolute log2‐fold change >1.1 and FDR ≤ 0.05 were selected. The differential expression of the top miRNAs was assessed by qRT‐PCR, and the fold change of expression levels between two prognostic groups was evaluated using the 2^−ΔΔCt^ method.^34^

In the internal test cohort, the univariate analysis was performed for all covariates including age, gender, race, TNM stage, depth of invasion, close/positive surgical margins, perineural invasion, histologic grade, and the expression levels of selected miRNAs. To develop the best prognostic model, we repeated the cross‐validation procedure 1000 times by randomly segregating data into ~70% training set and ~30% testing set. We further considered variables that were included in the backward‐stepwise selection Cox model on time‐to‐death with *p*‐value ≤ 0.1 more than 10% of the times out of the 1000 cross‐validations. Different Cox models with different sets of selected variables were constructed and the c‐indexes calculated. The model with the best performance (highest c‐index) was selected as the final prognostic model. Using the multiple logistic regression model, we further constructed a receiver‐operating characteristic (ROC) curve and calculated the area under the curve (AUC) for the internal test cohort using the same set of variables in the final prognostic model.

Using the final prognostic model derived from the internal test cohort, we generated the AUC for the internal and external validation cohorts. We also calculated the mortality risk score for every patient in the internal test and internal and external validation cohorts by summing the expression values of the selected miRNAs and covariates weighted by the regression coefficients obtained from the multivariate Cox regression analyses as previously described.^35^ Based on the individual mortality risk score, the patients were first stratified into higher vs lower mortality risk groups, and then further stratified into high vs moderately‐high vs moderately‐low vs low risk groups. Kaplan‐Meier curves were generated for the four mortality risk groups for the time‐to‐disease related death. Statistical analyses were conducted using *R* and *p* < .05 was considered statistically significant.

## 3 RESULTS

### 3.1 Subject characteristics

The demographic and clinicopathologic characteristics of the internal test and validation cohorts, as well as external validation cohorts are shown in Table 1 (total n = 551, 17 subjects excluded due to undetermined Ct value of the RNU6‐6p control in the qRT‐PCR analysis). Our study consists of patients diagnosed with OSCC between 1995 and 2012, who were clinically staged prior to the AJCC eighth modification. We restaged all internal patients with available pathology information using the AJCC 8 staging criteria. Approximately 1% of patients in the internal test cohort and internal validation cohorts were upstaged from pathologic stage I to II. There were no subjects upstaged from pathologic stage II to III. Our results are less than that reported by Cramer et al,^7^ in which 8% of pathologic stage I patients changed to stage II and 5% changed to stage III in 39 361 OSCC patients using the new staging system. Our study only included patients who underwent surgery with curative intent without elective neck dissection and/or adjuvant chemo/radiotherapy. Decision for surgical treatment alone is made partially based on favorable histologic findings (ie, lesser extent of tumor invasion of the underlying tissues), which may explain the lower proportion of patients upstaged using the AJCC 8 staging criteria.

**Table 1:** Clinical and pathologic characteristics of the patients included in this study

	Deep sequencing (n = 91)^a^		Internal test cohort (n = 191)^a^		Internal validation cohort (n = 101)		External validation cohort (n = 259)	
	Death^b^	Survival^c^	Death	Survival	Death	Survival	Death	Survival
Patients	n = 21	n = 70	n = 50	n = 141	n = 21	n = 80	n = 77	n = 182
Age	*P* = .236		*P* = .106		*P* = .672		*P* < .001*	
Mean (range)	70 (46‐89)	66 (33‐89)	68 (43‐89)	64 (41‐89)	69 (43‐88)	67 (33‐88)	72 (30‐97)	64 (25‐94)
Gender	*P* = .455		*P* = 1		*P* = .472		*P* = .855	
Female	11 (52%)	29 (41%)	22 (44%)	63 (44%)	8 (38%)	38 (47%)	27 (35%)	60 (32%)
Male	10 (47%)	41 (58%)	28 (56%)	78 (55%)	13 (61%)	42 (52%)	50 (64%)	122 (67%)
Race/Ethnicity	*P* = .002*		*P* < .001*		*P* = .008*		*P* = .383	
White non‐Hispanic	11 (52%)	46 (65%)	24 (48%)	94 (66%)	8 (38%)	51 (63%)	60 (78%)	146 (83%)
White Hispanic	3 (14%)	19 (27%)	12 (24%)	37 (26%)	8 (38%)	20 (25%)	0 (0%)	3 (1%)
Black non‐Hispanic	3 (14%)	2 (2%)	10 (20%)	3 (2%)	5 (23%)	3 (3%)	0 (0%)	1 (0.5%)
Black Hispanic	4 (19%)	0 (0%)	4 (8%)	0 (0%)	0 (0%)	0 (0%)	0 (0%)	1 (0.5%)
Asian	0 (0%)	3 (4%)	0 (0%)	7 (4%)	0 (0%)	6 (7%)	16 (21%)	23 (13%)
TNM Stage	*P* = .188		*P* = <.001*		*P* = .007*		*P* = .138	
Stage I	14 (67%)	60 (86%)	27 (54%)	114 (80%)	9 (42%)	60 (75%)	60 (78%)	158 (87%)
Stage II	7 (33%)	10 (14%)	23 (46%)	27 (19%)	12 (57%)	20 (25%)	17 (22%)	24 (13%)
Depth of Invasion	*P* = .549		*P* = 1.00		*P* = .059		*P* = .569	
<5 mm	20 (95%)	68 (97%)	49 (98%)	137 (97%)	18 (86%)	78 (97%)	1 (25%)	6 (55%)
>5‐10 mm	9 (5%)	2 (3%)	1 (2%)	4 (3%)	3 (14%)	2 (3%)	3 (75%)	5 (45%)
Close margins^d^	*P* = .573		*P* = .026		*P* = .339		*P* = .516^e^	
Not present	17 (81%)	50 (71%)	45 (90%)	105 (74%)	16 (76%)	68 (85%)	2 (50%)	9 (82%)
Present	4 (19%)	21 (29%)	5 (10%)	36 (26%)	5 (24%)	12 (15%)	2 (50%)	2 (18%)
Perineural Invasion	*P* = .111		*P* = 1.00		*P* = .026		*P* = .569^e^	
Not Present	21 (100%)	61 (87%)	45 (90%)	126 (89%)	15 (71%)	73 (91%)	1 (25%)	6 (55%)
Present	0 (0%)	9 (13%)	5 (10%)	15 (11%)	6 (29%)	7 (9%)	3 (75%)	5 (45%)
Histologic grading	*P* = .311		*P* < .001*		*P* < .001*		*P* = .139^e^	
Well‐differentiated	10 (48%)	44 (63%)	18 (36%)	91 (65%)	4 (19%)	47 (59%)	33 (43%)	98 (54%)
Moderately/poorly‐differentiated	11 (52%)	26 (37.14%)	32 (64%)	50 (35%)	17 (81%)	33 (41%)	44 (57%)	84 (46%)
Smoking status	*P* = .003*		*P* < .001*		*P* < .001*			
Never	9 (42%)	47 (68%)	18 (36%)	94 (68%)	6 (28%)	45 (56%)	na	na
Past	3 (14%)	15 (21%)	8 (16%)	29 (21%)	3 (14%)	32 (40%)	na	na
Current	9 (42%)	7 (10%)	24 (48%)	15 (10%)	12 (57%)	3 (3%)	na	na
Alcohol Abuse	*P* = .014*		*P* < .001*		*P* < .001*			
Never	13 (61%)	57 (82%)	26 (52%)	109 (78%)	8 (38%)	58 (72%)	na	na
Past	0 (0%)	4 (5%)	6 (12%)	14 (10%)	2 (9%)	10 (12%)	na	na
Current	8 (38%)	8 (11%)	18 (36%)	15 (10%)	11 (52%)	12 (15%)	na	na

*Note*: *Statistically significant difference (*p* < .05).

a Patients with deep sequencing data consists of 100 subjects from the internal subject pool; nine cases that did not pass the quality control were removed from the analysis.

b Patients who had cancer‐specific death in the 5‐year period following initial treatment.

c Patients who survived the first 5 years following initial treatment.

d Close (<5 mm) or positive surgical margins.

e Depth of invasion, close margins, and perineural invasion for external validation cohort was available for 15 subjects only.

Clinicodemographic variates known to be associated with poor prognosis include older age, male gender, and African‐American ethnicity.^4^, ^5^, ^6^, ^14^, ^16^, ^36^, ^37^, ^38^, ^39^, ^40^, ^41^ Tobacco and alcohol use are etiologic factors of oral cancer.^4^, ^5^, ^6^ TNM stage (includes the size of the lesion and the depth of invasion), histologic grading, close (<5 mm), or positive margins and the presence of perineural invasion are currently utilized as clinical measures to assess prognosis.^4^, ^5^, ^6^, ^14^, ^16^, ^36^, ^37^, ^38^, ^39^, ^40^, ^41^

In the internal test cohort, TNM stage II and moderate to poorly differentiated histologic grades were significantly associated with a higher risk for cancer‐specific mortality (Table 1). However, the depth of invasion, a new T‐classification modifier, was not prognostically significant by itself. Similarly, close margin (tumor within 0‐5 mm of the margin) and the presence of perineural invasion were not significantly associated survival outcome. While African‐American race/ethnicity showed an association with poor prognosis, due to the small number (only 22 Black non‐Hispanic out of 551 subjects), race/ethnicity was not included in further analysis. Information on tobacco and alcohol use was available only for the internal subjects. Statistical significance was observed for both the internal test and the validation cohorts with poor prognosis, in which patients who are current smokers were 7.5 times more likely (*p* < .001) to die of disease and those who abuse alcohol (4 or more drinks on any day or 8 or more drinks per week) were 4.6 times more likely (*p* < .001) to have cancer‐specific death within 5‐years of the initial cancer treatment. For those who are current smokers and also abuse alcohol, the odds ratio of cancer‐specific death within 5 years increased to 13.7 (*p* < .001).

### 3.2 Signature microRNA discovery

**Figure 1:**
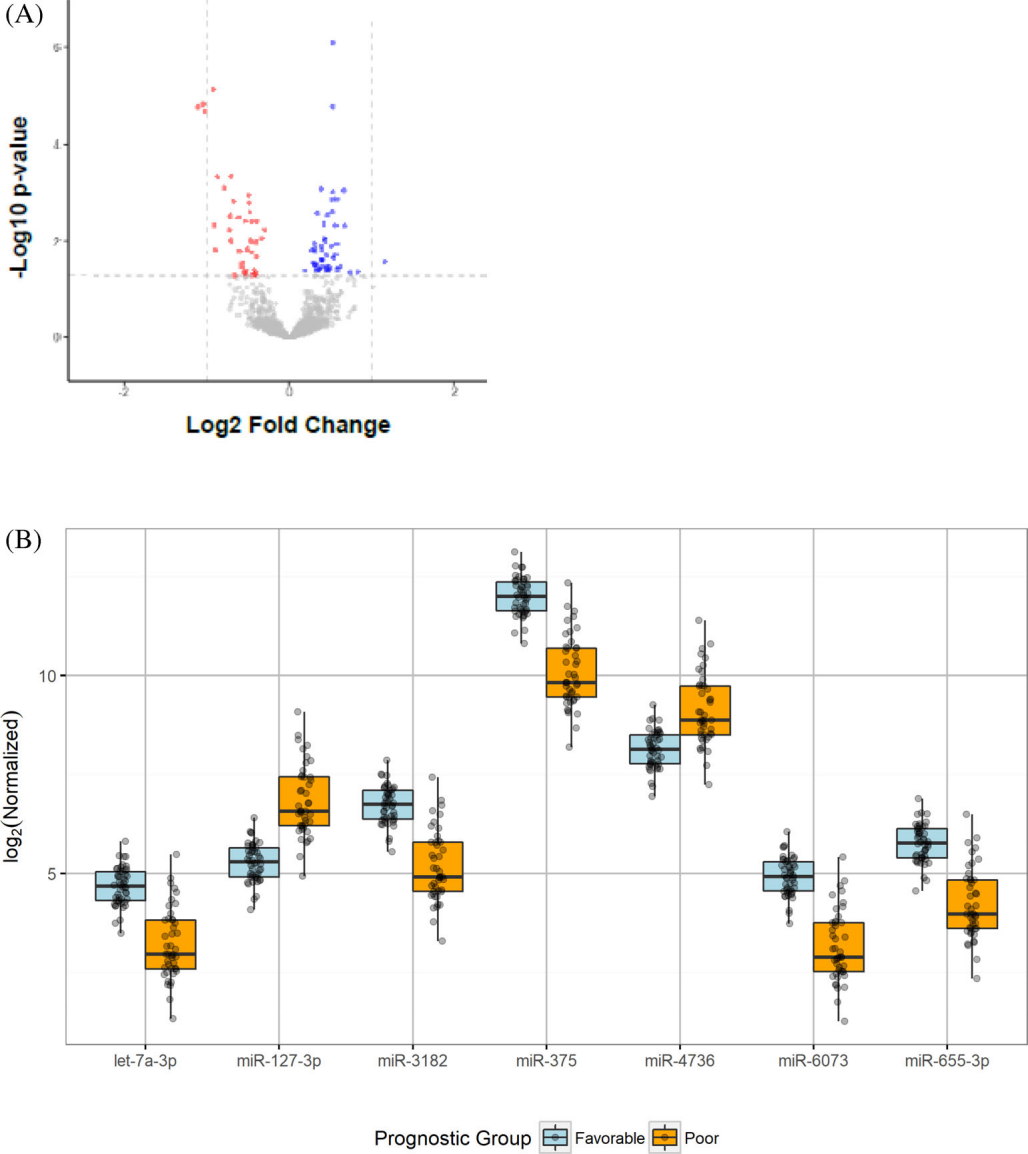
Deep sequencing analysis. (A) Volcano plot demonstrating over and underexpressed miRNAs between the poor prognosis group (cancer‐specific mortality) vs favorable prognosis group (5‐year survival following the initial treatment). (B) Boxplot representing the average expression (post‐normalization) of top seven microRNAs that demonstrate significant differential expression between poor vs favorable prognostic groups [Color figure can be viewed at wileyonlinelibrary.com]

Deep sequencing was performed on the 100 internal subjects using the EdgeSeq WT‐miRNA assay that targets 2083 miRNAs. The number of reads of each miRNA detected in the samples ranged from 0 to 1 306 955. When comparing between subjects with cancer‐specific death/recurrence (poor prognosis) vs those who survived 5 years (favorable prognosis), 365 miRNAs had significant log2‐fold change between two prognostic groups. Volcano plot of over and underexpressed miRNAs is shown in Figure 1A. Of these, 13 miRNAs had an absolute log2‐fold change >1.1 (ranging from 1.1 to 1.9) and FDR ≤ 0.05. Nine out of 13 miRNAs (miRNA‐127‐3p, 4736, 655‐3p, 6073, 3182, 381‐3p, 375, 378, and let‐7a‐3p) had commercially available miRNA probes and were further tested using qRT‐PCR in all 200 internal test subjects. Two miRNAs (miRNA‐381‐3p and 378b) had undetermined Ct‐values (>40 cycles) in more than half of the internal test cases and were excluded from further analysis. This is most likely due to the higher sensitivity of the deep sequencing analysis compared to that of qRT‐PCR. Nine cases with undetermined Ct value for the control RNU6‐6p were also excluded (n = 191). The potential prognostic roles of the selected seven miRNAs were investigated through a literature search. A boxplot demonstrating differential expression of the top seven miRNAs between the poor vs favorable prognostic groups obtained from deep sequencing analysis is shown in Figure 1B.

### 3.3 Prognostic model construction

The univariate analysis was performed on all clinicodemographic covariates and the top seven miRNAs selected from the deep sequencing in the internal test cohort. Those variables with “correct” directional change (ie, older age with positive univariate coefficient, etc.) were selected and subjected to the 1000 cross‐validations. The top six covariates selected over 100 times are shown in Table 2. Stepwise Cox models on time‐to‐death were constructed with the six covariates and the c‐index calculated (Table 3). The highest c‐index was observed in the model consisting of the top five covariates (c‐index of 0.816 with a SD of 0.046) and selected as the final prognostic model. The top five covariates include three miRNAs (miRNA‐127‐3p, 4736, 655‐3p) and two clinical variable (TNM stage and histologic grading). The normalized mean Ct‐values of three selected miRNAs and the fold change between the two prognostic groups is shown in Table 4.

**Table 2:** The 1000 cross‐validations to identify prognostically important variables

Variables	Times selected
miRNA‐127‐3p	996
miRNA‐655‐3p	930
TNM Stage	846
miRNA‐4736	441
Histologic grading	221
Let‐7a‐3p	104

*Note*: Those that were selected over 100 times were considered for further analysis.

**Table 3:** Final prognostic model selection with the prognostic variables selected from the 1000 cross‐validations using the c‐index calculation (mean and *SD*)

Models	c‐Index mean (*SD*)
[1] = c(miRNA‐127‐3p)	0.715 (0.056)
[2] = c(miRNA‐127‐3p, miRNA‐655‐3p)	0.739 (0.052)
[3] = c(miRNA‐127‐3p, miRNA‐655‐3p, TNM Stage)	0.792 (0.046)
[4] = c(miRNA‐127‐3p, miRNA‐655‐3p, TNM Stage, miRNA‐4736)	0.809 (0.049)
*[5] = c(miRNA‐127‐3p, miRNA‐655‐3p, TNM Stage, miRNA‐4736, Histologic grading)*	*0.816 (0.047)*
[6] = c(miRNA‐127‐3p, miRNA‐655‐3p, TNM Stage, miRNA‐4736, Histologic grading, let‐7a‐3p)	0.809 (0.502)

**Table 4:** Normalized mean expression levels of selected miRNAs obtained by qRT‐PCR and the fold change between those who had cancer‐specific death vs those who survived 5‐years following initial surgical treatment

Mean normalized Ct‐value				
miRNAs	Cancer‐specific mortality group	5‐year survival group	Fold‐change^a^	*P*‐value^b^
miRNA‐127‐3p	3.10	3.95	1.79	.001
miRNA‐4736	7.61	8.44	1.77	.004
miRNA‐655‐3p	12.16	11.65	0.71	.401

a Fold change calculated as 2^−ΔΔCt^.

b *p*‐value is based on the two‐sample *t*‐test comparing the mean normalized Ct values.

**Figure 2:**
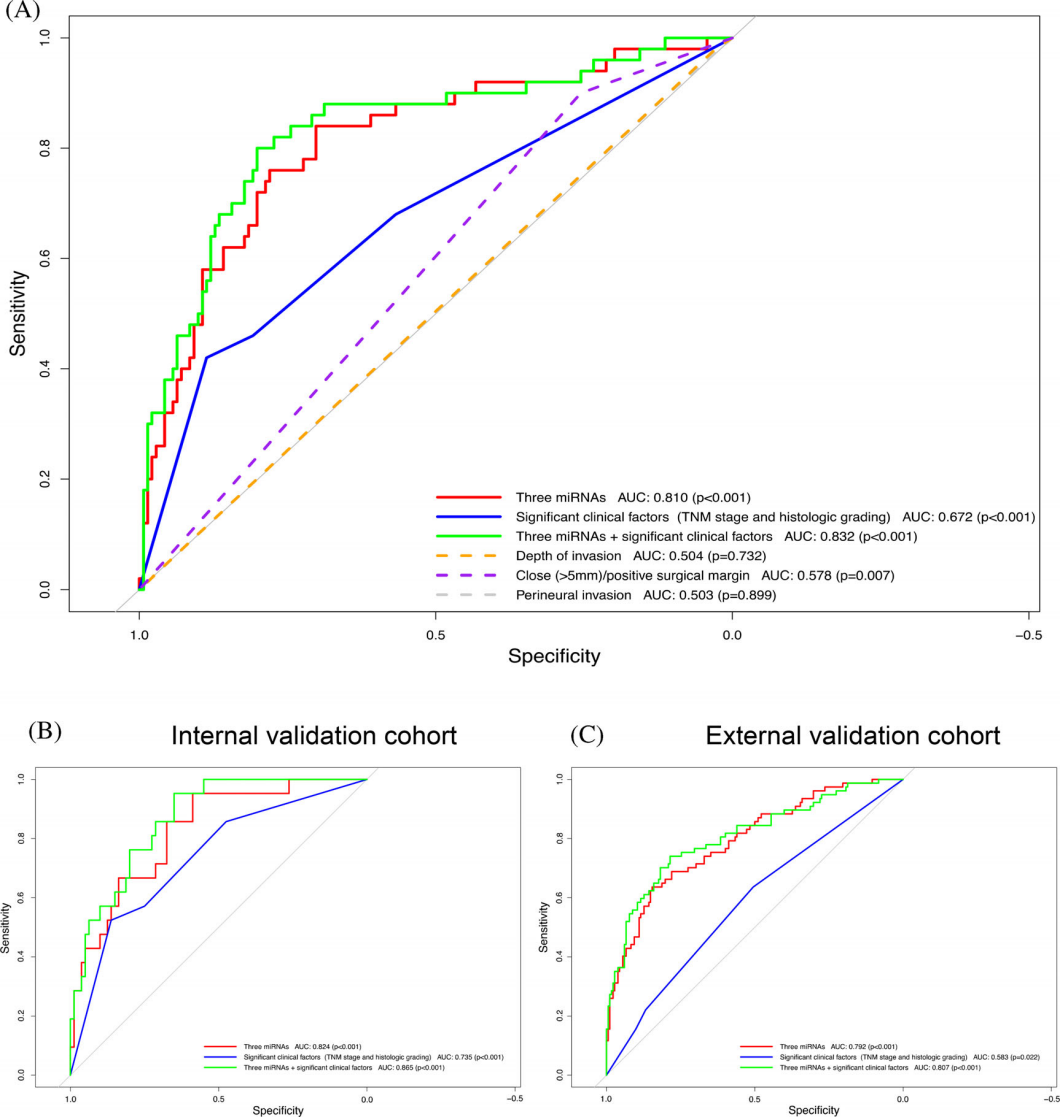
The ROC of the 5‐plex miRNA‐based prognostic marker panel, compared to clinical prognostic indicators alone and miRNAs alone. MicroRNAs include miRNAs‐127‐3p, 4736 and 655‐3p. The clinical prognostic factors include TNM stage I vs II and histologic grading, well vs moderately/poorly differentiated. The dashed lines in A represent some of clinically utilized prognostic factors including depth of invasion, close (>5 mm) or positive margins and perineural invasion. (A) Internal test cohort, (B) internal validation cohort, and (C) external validation cohort [Color figure can be viewed at wileyonlinelibrary.com]

Cox proportional hazards regression analysis of the top five prognostic variables was performed (Table 5), and the logistic regression analysis was conducted to construct an ROC curve and calculate the AUC in the internal test cohort (Figure 2). The AUC of the ROC curve with the miRNA‐based 5‐plex marker panel was 0.83 (95% CI: 0.76, 0.90; *p* < .001). The clinical prognostic indicators alone, including TNM stage and histologic grading, had an AUC of 0.67 (*p* < .001), demonstrating that the 5‐plex prognostic marker combining the signature miRNAs together with the existing clinical predictive modality significantly increase the prognostic power. The depth of invasion and perineural invasion had an AUC slightly higher than 0.50, although not significant. The close (<5 mm) or positive surgical margin showed some prognostic value (AUC of 0.58, *p* = .007).

**Table 5:** Cox proportional hazards regression analysis “time‐to‐death” for five covariates

	Regression coefficient	Hazard ratio	SE	*P*‐value
TNM Stage	0.897	2.452	0.327	.006*
Histologic Grade	0.385	1.469	0.345	.265
miRNA‐127‐3p	−0.738	0.478	0.187	.00008*
miRNA‐655‐3p	0.122	1.129	0.043	.0049*
miR‐4736	−0.265	0.767	0.141	.060

* Statistically significant difference (*p* < .05).

**Figure 3:**
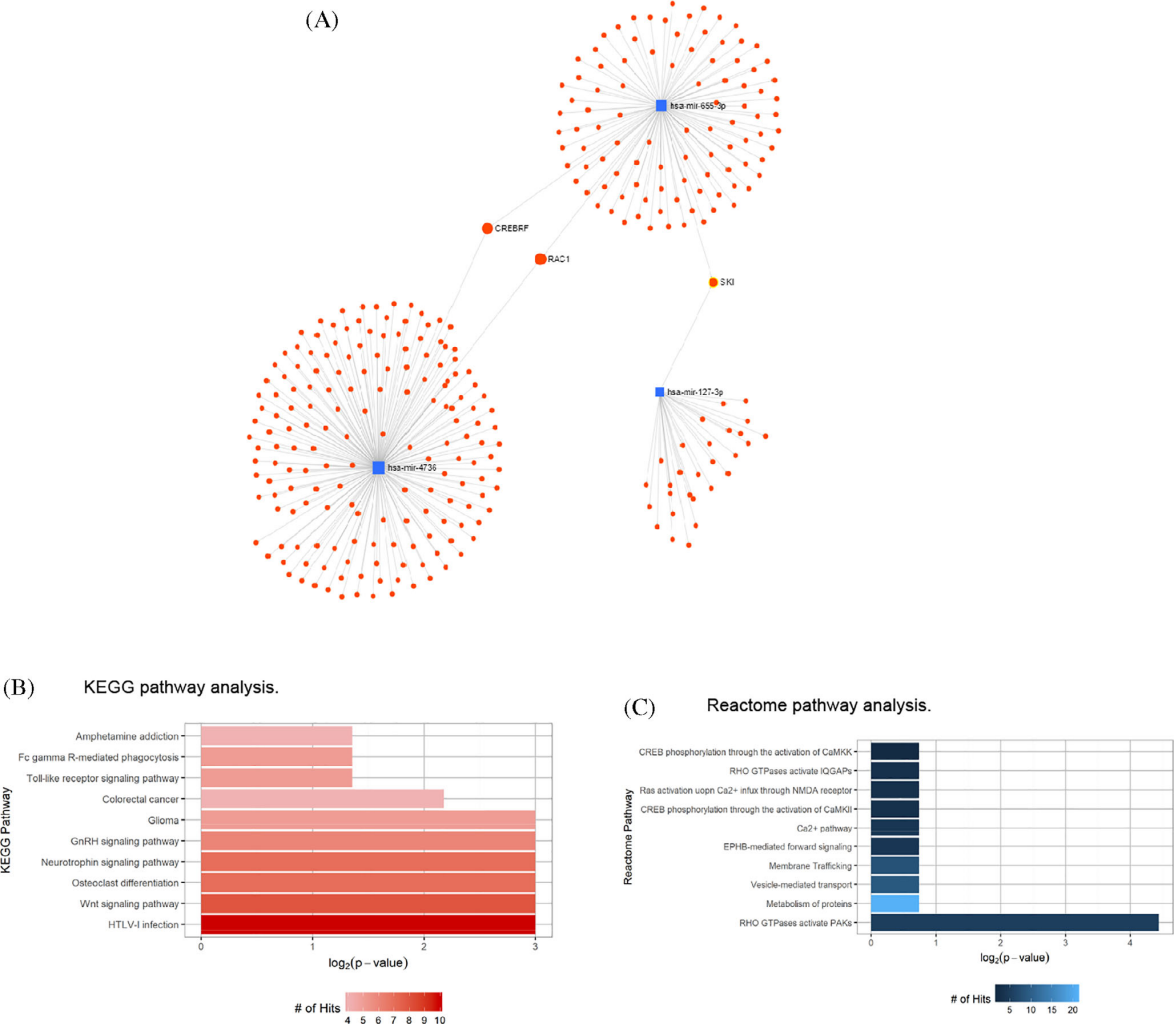
Network visualization and functional analysis. (A) Interaction networks of the prognostic miRNAs (miRNA‐127‐3p, 4736, and 655‐3p), the target genes and their involved pathways in the early‐stage oral cancer patients who died of disease within 5 years following initial treatment (poor‐prognosis group). The SKI proto‐oncogene, Rac family small GTPase 1 (RAC1) and CREBRF were identified as the network hubs. (B) Selected KEGG and (C) Reactome pathway analysis of the gene targets of selected miRNAs. Dysregulation of cancer‐related pathways involving RHO GTPases, Wnt/β‐cantenin, Ras, and toll‐like receptor (TLR) was identified in association with the miRNAs [Color figure can be viewed at wileyonlinelibrary.com]

Network visualization and functional analysis was performed as shown in Figure 3. MicroRNA‐655‐3p has a putative tumor suppressor role and downregulation of its expression level is observed in the poor prognosis group. On the other hand, miRNAs‐127‐3p and 4736 promote tumor progression and metastasis; higher levels are associated with increased risk for cancer‐specific mortality. Combined, these three miRNAs are involved in Ras activation, which is an important component of the signal transduction pathways to initiate cell growth and differentiation.^42^ These miRNAs also modulate toll‐like receptor (TLR) signaling pathways.^43^ TLRs play a crucial role in adaptive immune response and dysregulation of the pathway can lead to aberrant TLR activation, which in turn triggers NF‐κB signaling activation and overexpression of inflammatory cytokines such as IL‐1β, TNFα, and IL‐6, resulting in tumor cell proliferation and invasion.^43^

### 3.4 Mortality risk score calculation

The final prognostic model consisted of miRNAs‐127‐3p, 4736, 655‐3p, TNM stage, and histologic grade. Using this model, a prognostic risk score was calculated, which included the five covariates, each weighed by relative contribution: Mortality risk score = (−0.7 × expression value of miRNA‐127‐3p) + (−0.3 × expression value of miRNA‐4736) + (0.1 × expression value of miRNA‐655‐3p) + (0.9 × 0 for TNM stage I; 1 for TNM stage II) + (0.4 × 0 for well‐differentiated; 1 for moderately/poorly differentiated), in which miRNA expression level is the ΔCT value of each miRNA. The prognostic score was calculated for each patient in the internal test cohort and the patients were stratified into high vs low‐risk based on the score. The mortality risk score ranged from −2.40 to 2.96 (mean = 0). A higher score was considered to be associated with greater risk for cancer‐specific mortality. Using the mean risk score as the cutoff point, the morality risk score model was able to identify patients at greater risk for cancer‐specific mortality, with an overall predictive accuracy of 0.72, and sensitivity and specificity of 88% and 66%, respectively.

### 3.5 Internal and external validation

Using the final model driven from the internal test cohort, an ROC curve was generated for the internal validation cohort (n = 101). The AUC of the internal validation cohort (0.87, *p* < .0001) was slightly higher than that of the internal test cohort (0.83 for the internal test cohort, *p* < .0001). For the external validation cohort (n = 259), the AUC was 0.81 (*p* < .0001). The ROCs for the internal and external validation cohorts are shown in Figure 2. In the internal validation cohort, the mortality risk score ranged from −3.1 to 2.6, and in the external validation cohort, the mortality risk score ranged from −4.5 to 3.4. Using 0 as the cutoff, the risk score formula was able to correctly stratify 76% of patients who died of cancer as higher risk (16 out of 21) in the internal validation cohort, with predictive accuracy of 0.73. In the external validation cohort, 65% of patients who died of the disease were classified as higher risk (50 out of 77), with a predictive accuracy of 0.81. Overall, for all three cohorts combined, the miRNA‐based marker panel had a predictive accuracy of 0.76 with a sensitivity of 74% and a specificity of 77% in identifying those at high‐risk for cancer‐specific death.

### 3.6 Prognostication based on the risk scores

Since the goal of the study is to accurately identify patients at high‐risk for cancer‐specific mortality among those assigned to early TNM stage, and also to provide practical guidance for the clinicians to assess prognosis, we further stratified patients into four risk categories based on the risk score as shown in Table 6. When the three cohorts were combined, a risk score of 2 or higher was associated with a significantly high‐risk for cancer‐specific mortality and recurrence, in which 94% of subjects in this score range died of the disease with a median survival time of 11 months (HR of high‐risk compared to low‐risk = 23, *p* < .001). In comparison, those with a risk score of less than 0 were considered to be low‐risk with 11% of subjects dying of the disease with a median survival time ≥ 60 months. The Kaplan‐Meier curves for the high, moderately‐high, moderately‐low and low‐risk groups stratified based on the risk scores are shown in Table 6.

**Table 6:** Four risk categories (high vs moderately‐high vs moderately‐low vs low) based on the cancer‐specific mortality risk score and the Kaplan‐Meier curve

	Risk scores	Median time to death^a^	Median time to recurrence^a^	Cancer death (%)	5‐year survival (%)	HR^b^
Internal test cohort	≥2	11	2	89	11	44*
	1 to <2	38	13	62	38	16*
	0 to <1	≥60	≥60	35	65	7*
	<0	≥60	≥60	6	94	1
Internal validation cohort	≥2	12	1	100	0	26*
	1 to <2	14	7	67	33	15*
	0 to <1	≥60	≥60	22	78	3
	<0	≥60	≥60	8	92	1
External validation cohort^c^	≥2	27	27	100	0	16*
	1 to <2	22	22	88	12	13*
	0 to <1	36	36	59	41	6*
	<0	≥60	≥60	15	85	1
*All three cohorts combined*						
Risk categories						
High risk	≥2	11	6	94	6	23*
Moderately‐high risk	1 to <2	22	14	71	29	11*
Moderately‐low risk	0 to <1	≥60	46	42	58	5*
Low risk	<0	≥60	≥60	11	89	1

*Note*: *P < .00001.

a Median time‐to‐death and time‐to recurrence in months.

b HR: hazard ratio of cancer‐specific death of each risk category compared to the low‐risk group (risk scores<0 as the reference group).

c Median time‐to‐recurrence for the external cohort based on 82 subjects with recurrence information obtained during the review of pathology reports.
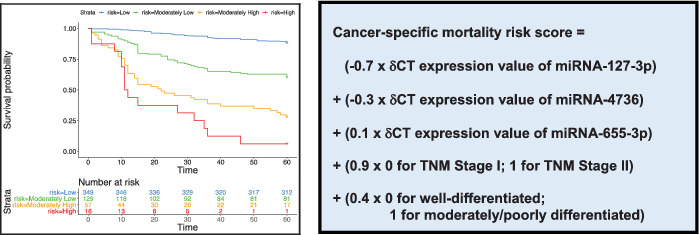

## 4 DISCUSSION

Currently, there is no clinical modality to identify patients at high‐risk for cancer‐specific death among those assigned to early‐stage OSCC (TNM stages I and II). Because 50% of oral cancer patients are in early‐stage at the time of diagnosis,^2^ a window of opportunity exists in which proper prognostication and subsequent decisions for additional treatment can dramatically improve the 5‐year survival of patients with this deadly disease.

We have previously identified two prognostic miRNAs (miRNA‐375 and 214‐3p) in patients who had surgery with or without neck dissection in a smaller subject pool (n = 100).^35^ The two miRNA markers with age and gender was able to identify patients at high‐risk for cancer‐specific death vs those who will have cancer‐free 5‐year survival. Here, we designed a new study with 568 early‐stage oral cancer patients who had surgery alone as the treatment. The miRNAs‐127‐3p, 4736, 655‐3p, together with the AJCC 8 TNM staging system, and histologic grade demonstrated significant prognostic power. Our miRNA‐based 5‐plex marker panel is optimized to differentiate between those who develop recurrence and die of cancer vs those who survive despite loco‐regional recurrence, in addition to discriminating between those who have cancer‐specific death vs those who have 5‐year cancer‐free survival.

We discovered miRNA signatures using next‐generation sequencing, and validated the prognostic power of miRNAs using qRT‐PCR. We then built a 5‐plex prognostic model consisting of miRNAs and clinical covariates. The mortality risk score formula was driven from the final prognostic model to serve as a quantifiable risk assessment modality that can be readily applied in a clinical setting. To construct a robust prognostic marker panel, the prognostic model and the risk score formula driven from the internal test cohort (n = 191) were validated in an independent internal validation cohort (n = 101) as well as an external validation cohort (n = 259). The external validation cohort consisted of a heterogeneous population in the Northeastern and Midwestern US and Hawaii to ensure generalizability of our findings across populations. The AUC of the ROC curve with the miRNA‐based 5‐plex marker panel was 0.83 (*p* < .001), 0.87 (*p* < .001), and 0.81 (*p* < .001) in internal test, internal validation and external validation cohorts, respectively, demonstrating uniformly significant prognostic value.

When the early‐stage OSCC patients were stratified into two risk groups, higher (≥0) vs lower (<0) risk, the overall predictive accuracy, sensitivity, and specificity were 0.76, 74%, 77%, respectively. To provide practical clinical guidance, we further stratified patients into four risk categories based on the risk scores; high (≥2) vs moderately‐high (2 to ≥1) vs moderately‐low risk (1 to ≥0) vs low‐risk (<0), in which the rate of cancer‐specific death changes from 94% to 71% for high and moderately‐high risk groups, and from 42% to 11% for the moderately‐low to low risk groups. High and moderately‐high risk scores were also associated with a shorter median time‐to‐recurrence of 6 and 14 months, respectively. The early‐stage oral cancer patients with high or moderately‐high risk scores (score ≥ 1) may benefit from active intervention, that is, neoadjuvant therapy, elective neck dissection, irradiation, and so on, as opposed to those with moderately‐low risk scores (1 to ≥0), who may be placed under vigilant observation for signs of recurrence following initial surgery.

Larger tumor size and increased depth of tumor invasion (TNM stage II; size ≤ 2 cm and DOI > 0.5 but ≤1.0 cm, or size 2‐4 cm and DOI ≤ 1.0 cm) and histologic evidence of moderately/poorly differentiated cancer are currently utilized prognostic factors to predict clinical outcome. Indeed, these two clinical factors had some prognostic value with an AUC of 0.67 (*P* < .001). When combined with the three miRNAs, the 5‐plex prognostic marker panel demonstrated significantly greater prognostic power with an AUC of 0.83. The presence of unfavorable histology such as close (<5 mm) or positive surgical margins and evidence of perineural invasion had marginal prognostic value. Most patients included in our study had “close margins” rather than “tumor at the margin,” which explains the limited prognostic value of close/positive margin. The DOI alone did not have prognostic significance. It becomes significant only when the DOI is incorporated with the size of the tumor into the TNM stage.

In terms of the miRNAs included in our final model, miRNA‐127‐3p functions as an oncogene and promotes the migration and invasion of tumor cells. In glioblastoma, miRNA‐127‐3p targets and inhibits SEPT7, which is a negative regulator of cell migration and invasion.^44^ Upregulation of miRNA‐127‐3p was observed in tumor initiating cells in lung carcinoma and also in circulating blood in breast cancer patients compared to that of healthy individuals.^45^, ^46^ Increased expression levels of serum miRNA‐4736 was observed in patients with various sarcomas. Hence, miRNA‐4736 has an oncogenic role, and a step‐by‐step increase in expression levels correlates with disease severity (lowest in healthy individuals, moderate in benign tumors, and highest in sarcoma patients).^47^ miRNA‐655‐3p regulates E‐cadherin expression and inhibits β‐catenin signal pathway, thereby functioning as a tumor suppressor in hepatocellular carcinoma.^48^ It also halts tumor invasion and metastatic dissemination in various cancer types.^49^

This study has a modest sample size due to strict subject inclusion and exclusion criteria to select only those in the early TNM stage of oral cancer who had surgery alone as the treatment. A number of early‐stage OSCC patients have had elective neck dissection performed due to the possibility of occult lymph node metastasis and these cases were excluded from the study. However, the sample size was adequate in achieving significance in the prognostic power of each cohort. The prognostic value is slightly lower for the external validation cohorts compared to that of the internal cohorts, which is bound to differ due to different geographic locations with different clinical practices.^50^

In sum, we evaluated a miRNA‐based risk stratification modality for early‐stage oral cancer patients. The TNM stage and the histologic grading are readily available clinical information, and the miRNA expression levels can be obtained with relative ease using qRT‐PCR in the formalin‐fixed paraffin‐embedded tumor biopsy tissue. Thus, the prognostic risk score calculation can be performed as a part of the pathology work‐up. We have plans to prospectively assess the prognostic value of this miRNA‐based model in a large‐scale multicenter setting to provide the highest level of evidence supporting the clinical validity and usefulness of the biomarker. We also intend to evaluate the efficacy of various treatment regimens (elective neck dissection, irradiation, combination of neck dissection, and radiotherapy in addition to surgery) in patients stratified into high and low‐risk categories using the miRNA‐based prognostic model as the next step. The study results can then be utilized to guide decision‐making in treatment selection and make risk‐adjusted therapies possible for patients in early‐stage OSCC.
